# The spatial–temporal distribution and etiological characteristics of hand-foot-and-mouth disease before and after EV‑A71 vaccination in Kunming, China, 2017–2020

**DOI:** 10.1038/s41598-022-21312-2

**Published:** 2022-10-11

**Authors:** Meifen Wang, Tao Chen, Junchao Peng, Yunjiao Luo, Lijiang Du, Zhiying Lu, Jianzhu He, Chunli Liu, Quan Gan, Wei Ma, Zhikuan Cun, Qiongmei Zheng, Weiying Chen, Yonglin Chen, Mei Han, Guojun Liu, Jiwei Li

**Affiliations:** 1 Department of Infectious Diseases, Kunming Children’s Hospital, The Affiliated Children’s Hospital of Kunming Medical University, Institute of Pediatric Disease Research in Yunnan, Kunming, 650228 People’s Republic of China; 2 Department of Pathology, Kunming Children’s Hospital, The Affiliated Children’s Hospital of Kunming Medical University, Institute of Pediatric Disease Research in Yunnan, Kunming, 650228 People’s Republic of China; 3 Yunnan Key Laboratory of Children’s Major Disease Research, Kunming, People’s Republic of China; 4 Department of Stomatology, The Affiliated Hospital of Yunnan Normal University, Kunming, People’s Republic of China; 5 The First Department of Hepatopancreatobiliary Surgery, Affiliated Calmette Hospital of Kunming Medical University and The First People’s Hospital of Kunming City, Liver Transplantation Center of Organ Transplantation Institute of Yunnan Province, Kunming, People’s Republic of China; 6 Weather Modification Center of Yunnan Province, Kunming, People’s Republic of China; 7 Weifang Center for Disease Control and Prevention, Shandong, People’s Republic of China

**Keywords:** Infectious diseases, Disease prevention, Paediatrics, Public health

## Abstract

After vaccination with enterovirus 71 (EV-A71), the prevalence of hand-foot-and-mouth disease (HFMD) remained high, and the spatial–temporal distribution of enteroviruses changed. Therefore, it is essential to define the temporal features, spatial distributions, and epidemiological and etiological characteristics of HFMD in Kunming. Between 2017 and 2020, a total of 36,540 children were diagnosed with HFMD in Kunming, including 32,754 children with enterovirus-positive clinical samples. Demographic, geographical, epidemiological and etiological data of the cases were acquired and analyzed. Other enteroviruses replaced EV-A71, and the incidence of EV-A71 decreased dramatically, whereas coxsackievirus A6 (CV-A6) and coxsackievirus A16 (CV-A16) had substantial outbreaks in 2018 and 2019, respectively. The major and minor peaks all extended for 2–4 months compared to before vaccination with the EV-A71 vaccine. From 2019 to 2020, CV-A6, as the predominant serotype, showed only a single peak. Although a high incidence of HFMD was observed in Guandu, Chenggong and Xishan, the annual incidence of different enterovirus serotypes was different in different regions. In 2017, other enteroviruses were most prevalent in Shilin. In 2018, CV-A16 and CV-A6 were most prevalent in Luquan and Shilin, respectively. In 2019, CV-A16 was most prevalent in Jinning. In 2020, CV-A6 and coxsackievirus A10 (CV-A10) were most prevalent in Luquan and Shilin, respectively. Meanwhile, the epidemic cycle of CV-A6 and CV-A16 was only 1 year, and CV-A10 and other enteroviruses were potential risk pathogens. The spatial and temporal distribution of HFMD varies at different scales, and the incidence of HFMD associated with different pathogens has obvious regional differences and seasonal trends. Therefore, research on multivalent combined vaccines is urgently needed, and proper preventive and protective measures could effectively control the incidence of HFMD-like diseases.

## Introduction

Since 1997, hand-foot-and-mouth disease (HFMD) has been primarily prevalent in the Asia–Pacific region, including China, Japan, Singapore, Malaysia, Vietnam, South Korea, Thailand, Cuba, India and Cambodia. In mainland China, with an incidence of 37.01/100,000–205.06/100,000 people^1,2^, a total of 23.5 million HFMD cases were reported to the Chinese Center for Disease Control and Prevention (CDC) during 2000–2018^3,4^ and showed a continuous upward trend in recent years. Previous studies have focused on the eastern coastal, densely populated and economically developed cities (including Shandong, Anhui, Jiangxi, Jiangsu, Zhejiang, Fujian, Guangdong Province, Beijing, Shanghai and Shenzhen municipality) and indicated the epidemiological characteristics of EV-A71 and CV-A16, which were considered the most common etiological agents of HFMD in the past two decades^5^. However, less is known about what causes the different characteristics of the descriptive epidemiology of HFMD between different areas^6–10^.

To control the HFMD epidemic, the inactivated monovalent EV-A71 vaccine has been wildly administered throughout China since 2016, and is highly effective against EV-A71-associated HFMD^11,12^, but the prevalence of HFMD induced by other serotypes remains high^13,14^. Meanwhile, due to the coronavirus disease 2019 (COVID-19) pandemic, public areas were closed, and quarantine of infected patients was imposed beginning in December 2019. We implemented measures of regional surveillance of epidemics and no physical contact with infected patients, which played an important role in reducing HFMD-like disease cases in 2020.

As a medium-sized developing inland city in Southwest China with a high altitude and complex terrain, Kunming urgently needs research on the temporal features, spatial distributions, and epidemiological and etiological characteristics of HFMD, with an in-depth analysis before and after the EV-A71 vaccination campaign and COVID-19 pandemic.

## Materials and methods

### Study area

Kunming is the largest city in Yunnan Province in Southwest China. Within an area of approximately 21,473 square kilometers, the city has a complex terrain consisting of mountains, hills, plains, basins and plateaus and a subtropical monsoon continental climate. The city administers 7 districts, 1 county-level cities, 3 counties and 3 autonomous counties, and has a population of 8.46 million people now, including 1,267,713 people aged 0–14, accounting for 14.98%.

Kunming Children’s Hospital is a unique tertiary care pediatric hospital in Yunnan Province that integrates medical treatment, scientific research, teaching, health care and rehabilitation, with over 1200 inpatient beds.

### Data collection

We collected surveillance data on the prevalence of HFMD from Kunming Children’s Hospital from 2017 to 2020, including sex, age, population classification, address, type of diagnosis and laboratory test results (confirmed by fluorescence quantitative real-time reverse transcriptase PCR (qRT‒PCR) in the clinical laboratory of the hospital) of 36,540 HFMD cases.

### Clinical definition

According to the guidelines of the National Health Commission of the People’s Republic of China, a clinical case of HFMD was defined as a patient with maculopapular or vesicular rash on the hands, feet, mouth, or buttocks, with or without fever. Severe HFMD patients had at least one of the following: cardiopulmonary collapse, pulmonary hemorrhage, pulmonary edema, encephalitis, aseptic meningitis, acute flaccid paralysis, myocarditis, or death.

### Pathogenic detection

After the preliminary clinical diagnosis of HFMD, the enterovirus genotypes were determined using qRT‒PCR for clinical specimens. Feces were collected from sampling cases and kept at − 80 °C until examination for enterovirus by qRT‒PCR. Viral RNA was extracted from the clinical specimens using the Viral RNA Mini Kit (QIAamp Viral RNA Mini Kit (50) 52,904 Qiagen, Germany) in accordance with the manufacturer’s instructions and was subjected to qRT‒PCR. Laboratory test results were divided into 6 categories: EV-A71 positive, CV-A16 positive, CV-A6 positive, CV-A10 positive and other enteroviruses positive and enterovirus negative.

### Vaccination criteria

The inactivated EV-A71 vaccine can be used for preventing HFMD caused by EV-A71 infection in children aged 6 months to 5 years. The basic immunization procedure is 2 doses with an interval of 1 month. It is recommended that vaccination be completed before the age of 12 months.

### Statistical analysis

Descriptive epidemiological methods were used to analyze the demographic (including age, sex and population classification), etiological (EV-A71, CV-A16, CV-A6, CV-A10 and other enteroviruses), temporal and spatial characteristics of HFMD by month.

SPSS 22.0 statistical software was used for statistical analysis. Basic data were analyzed by descriptive analysis, and the counting data were analyzed by the chi-square test. The alpha value α = 0.05 was used. The geographic distribution map of HFMD cases was drawn by MapInfo 10.0 spatial distribution software.

### Ethics statement

All the study procedures were reviewed and approved by the Ethics Committee of Kunming Children’s Hospital. Written consent was obtained from each study participant’s parents or guardians.

## Results

### Demographic characteristics

A total of 32,754 positive cases of HFMD were monitored from 2017 to 2020 in Kunming. The annual incidence rates were 71.58/100,000 at minimum and 163.24/100,000 at maximum, and the average incidence rate was 118.97/100,000 (Table 1). HFMD patients under 5 years of age predominated and accounted for 93.30%, 52.17% of HFMD patients were aged 0–3 years, and 41.13% were aged 3–5 years. The male-to-female ratio of HFMD patients was 1.46:1 (ranging from 1.43:1 to 1.49:1) and showed a steady trend. The highest proportion of classified HFMD cases was observed among children of different ages, accounting for 55.77%; the second highest proportion was observed among nursery children, accounting for 39.97%; and the proportion of school-age-children was small, accounting for 4.26% (Table 2).

**Table 1:** The spatial distribution of HFMD annual incidence of districts and counties in Kunming, 2017–2020.

Years	2017	2018	2019	2020
Number	4855	11,182	10,311	6406
Incidence	71.58/100,000	163.24/100,000	148.37/100,000	92.17/100,000
**Location**				
Anning	39.72	53.54	65.55	41.39
Chenggong	82.48	245.82	302.61	154.94
Dongchuan	31.10	65.49	51.93	37.54
Fumin	28.75	74.00	38.36	33.96
Guandu	176.27	384.51	344.32	204.89
Jinning	38.51	89.59	68.95	36.86
Luquan	14.97	78.17	45.33	81.02
Panlong	67.44	149.05	133.22	77.90
Shilin	1.52	11.37	9.02	5.26
Songming	76.92	167.99	156.58	73.39
Wuhua	75.50	154.86	125.78	102.87
Xishan	108.87	235.78	217.55	131.79
Xundian	19.27	70.68	52.14	32.24
Yiliang	21.37	60.74	59.76	17.01

**Table 2:** The epidemiological description of HFMD in Kunming, 2017–2020.

	Years	2017	2018	2019	2020	*P-*value*
	Total	4855	11,182	10,311	6406	
Age group						< 0.001**
0–3	Number	2508	6010	5255	3316	
	Percentage	51.7%	53.7%	51.0%	51.8%	
3–5	Number	2093	4458	4287	2632	
	Percentage	43.1%	39.9%	41.6%	41.1%	
≥ 6	Number	254	714	769	458	
	Percentage	5.2%	6.4%	7.5%	7.1%	
Gender						0.364***
Male	Number	2864	6694	6061	3825	
	Percentage	59.0%	59.9%	58.8%	59.7%	
Female	Number	1991	4488	4250	2581	
	Percentage	41.0%	40.1%	41.2%	40.3%	
Hosting classification						< 0.001**
Scatter children	Number	2559	6396	5783	3529	
	Percentage	52.7%	57.2%	56.1%	55.1%	
Childcare	Number	2141	4362	4085	2502	
	Percentage	44.1%	39.0%	39.6%	39.1%	
School	Number	155	424	443	375	
	Percentage	3.2%	3.8%	4.3%	5.9%	
**Location**						
Anning	Number	150	204	255	161	
	Percentage	3.1%	1.8%	2.5%	2.5%	
Chenggong	Number	281	868	1123	575	
	Percentage	5.8%	7.8%	10.9%	9.0%	
Dongchuan	Number	88	186	148	107	
	Percentage	1.8%	1.7%	1.4%	1.7%	
Fumin	Number	45	117	61	54	
	Percentage	0.9%	1.0%	0.6%	0.8%	
Guandu	Number	1584	3514	3250	1934	
	Percentage	32.6%	31.4%	31.5%	30.2%	
Jinning	Number	118	277	217	116	
	Percentage	2.4%	2.5%	2.1%	1.8%	
Luquan	Number	62	324	188	336	
	Percentage	1.3%	2.9%	1.8%	5.2%	
Panlong	Number	565	1255	1127	659	
	Percentage	11.6%	11.2%	10.9%	10.3%	
Shilin	Number	4	30	24	14	
	Percentage	0.1%	0.3%	0.2%	0.2%	
Songming	Number	253	573	559	262	
	Percentage	5.2%	5.1%	5.4%	4.1%	
Wuhua	Number	661	1360	1109	907	
	Percentage	13.6%	12.2%	10.8%	14.2%	
Xishan	Number	859	1869	1735	1051	
	Percentage	17.7%	16.7%	16.8%	16.4%	
Xundian	Number	91	336	249	154	
	Percentage	1.9%	3.0%	2.4%	2.4%	
Yiliang	Number	94	269	267	76	
	Percentage	1.9%	2.4%	2.6%	1.2%	
**Identification of enterovirus**						
EV-A71	Number	504	430	130	2	
	Percentage	10.4%	3.8%	1.3%	0.0%	
CV-A16	Number	629	3359	4866	263	
	Percentage	13.0%	30.0%	47.2%	4.1%	
CV-A6	Number	10	3728	3473	4537	
	Percentage	0.2%	33.3%	33.7%	70.8%	
CV-A10	Number	11	450	167	457	
	Percentage	0.2%	4.0%	1.6%	7.1%	
Other enteroviruses	Number	3701	3215	1676	1147	
	Percentage	76.2%	28.8%	16.3%	17.9%	

*Comparison of HFMD patients with age group, gender and hosting classification in 2017–2020 by chi-square test.

**Significant statistical differences, *P* < 0.001.

***No statistically significant differences, *P* > 0.05.

### Enterovirus serotype distribution

**Figure 1:**
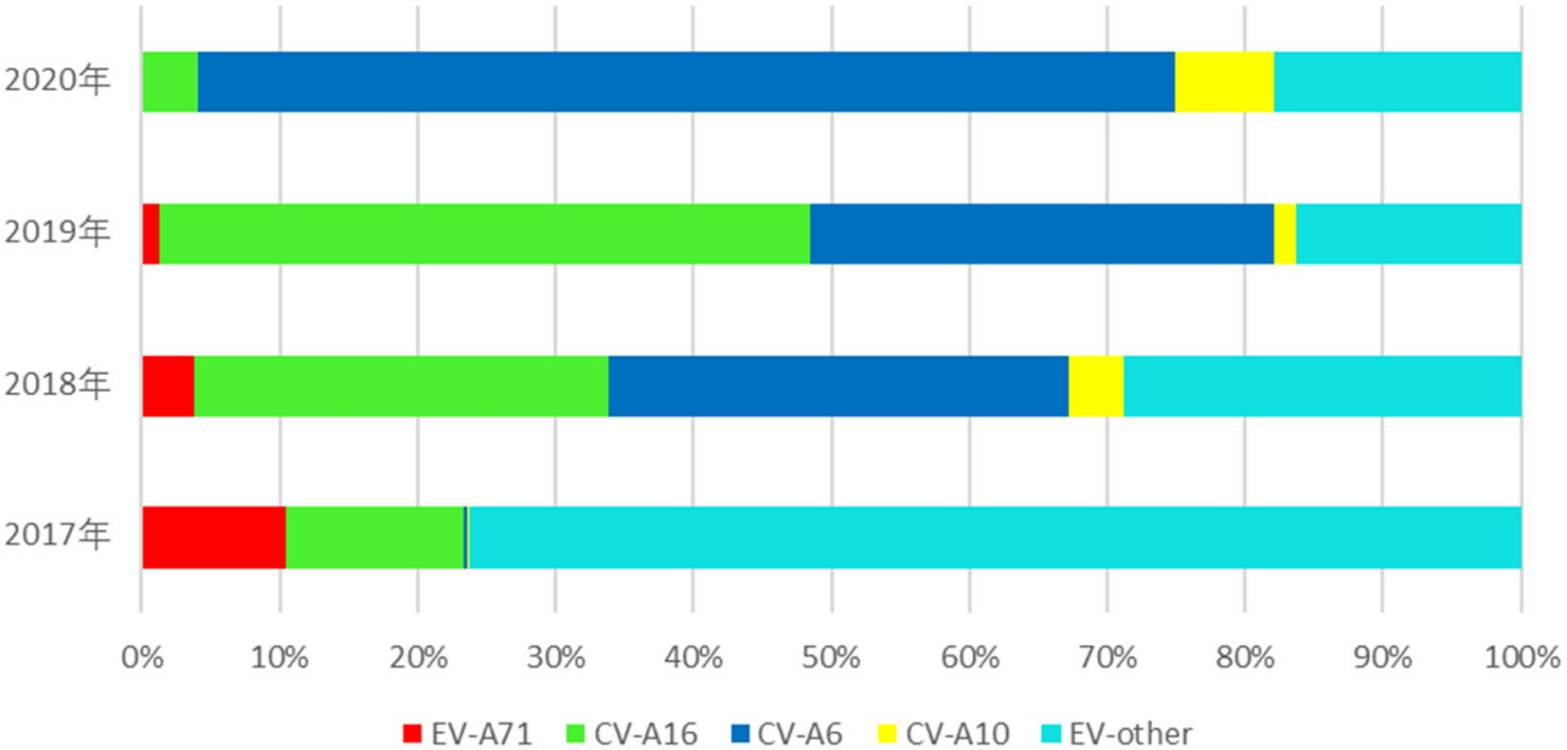
The annual percentages of positive enteroviruses of HFMD in Kunming, 2017–2020.

The numbers of serotypes of laboratory-confirmed cases fluctuated during 2017–2020, and we observed that there were two important time inflection points of change in enterovirus serotypes in 2017 and 2020. The annual percentages of EV-A71 were 10.4%, 3.8%, 1.3%, and only two cases from 2017 to 2020, respectively, which decreased continuously. The number of other enteroviruses and CV-A16 first trended upward and then decreased. Other enteroviruses increased substantially and accounted for 76.2% in 2017 but decreased yearly to 28.8% in 2018, 16.3% in 2019, and 17.9% in 2020. CV-A16 increased gradually and accounted for 30.0% and 47.2% in 2018 and 2019, respectively, but decreased dramatically to 4.1% in 2020. The numbers of CV-A6 and CV-A10 cases trended upward continuously; in particular, CV-A6 increased substantially and accounted for 33.3% and 33.7% in 2018 and 2019, respectively, and suddenly rose to 70.8% in 2020. The annual number of CV-A10 cases remained at a low level from 2017 to 2019, 0.2%, 4.0%, and 1.6%, respectively, but increased to 7.1% in 2020 (Table 2 and Fig. 1).

### Temporal characteristics

**Figure 2:**
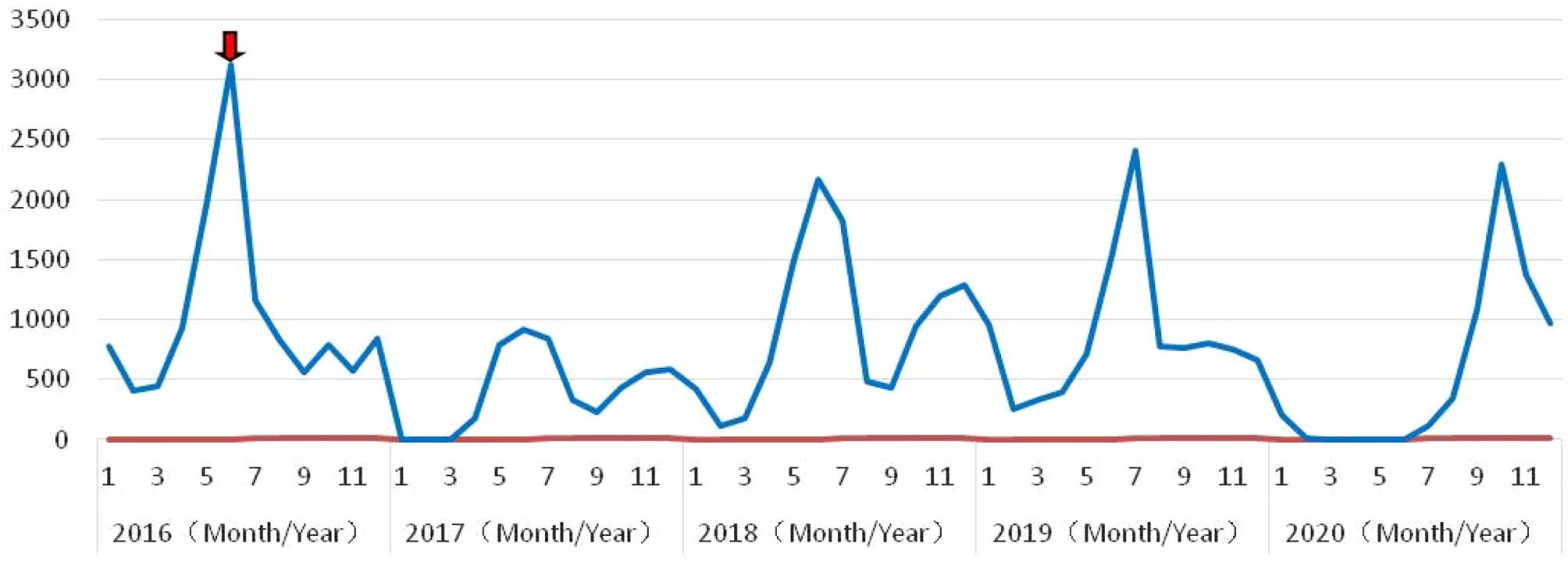
The temporal distribution with periodic peak of HFMD in Kunming, 2016–2020. The red arrow indicates that the local CDC began to vaccinate local children with EV-A71 vaccine in June 2016.

The annual incidence of HFMD in Kunming initially increased and presented a seasonal distribution with semiannual or annual peaks. One peak was observed in 2016 from April to June before vaccination with the EV-A71 vaccine. Two peaks were observed in 2017 and 2018, a major peak from April to August and another from September to the following February, and the peak value in 2018 was much higher than that in 2017. A single peak appeared in 2019 and 2020, and although the peak values were similar, they showed obvious differences. The peak in 2019 was followed by a plateau until the following February; in 2020, there was only one peak from July to the following February (Fig. 2).

### Spatial characteristics

**Figure 3:**
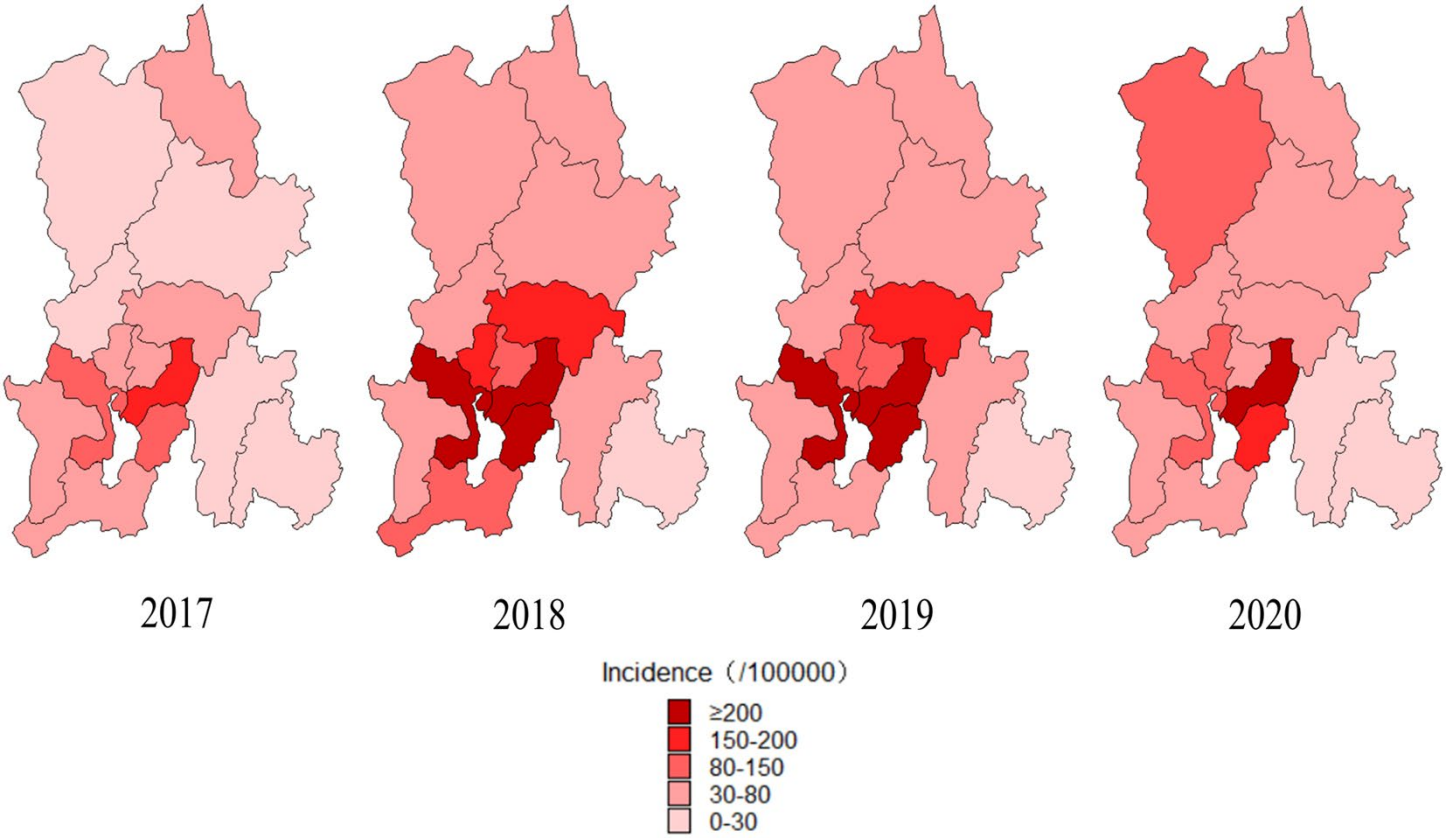
The spatial distribution map of HFMD annual incidence of districts and counties in Kunming, 2017–2020. The number of HFMD cases mainly concentrated in Guandu, Xishan, Wuhua, Panlong and Chenggong, and the high incidence of HFMD areas were Guandu, Chenggong and Xishan. This figure was drawn by MapInfo 10.0 spatial distribution software [url = http://www.i-mapinfo.com]MapInfo[/url].

During 2017–2020, 7 districts, 1 county-level city, 3 counties and 3 autonomous counties in Kunming reported the occurrence of HFMD. The spatial distribution of the incidence of HFMD was heterogeneous at the county level, ranging from 1.52/100,000 (Shilin) to 384.51/100,000 people (Guandu). The counties with the highest incidence of HFMD were Guandu, Chenggong, Xishan, Songming, Wuhua and Panlong. In some regions, such as Luquan, the incidence increased gradually from 14.97/100,000 in 2017 to 78.17/100,000 in 2018 and 81.02/100,000 in 2020. Overall, the area with the highest incidence was the core area of Kunming, which has a concentrated population and prosperous economy (Table 1 and Fig. 3).

### Spatial–temporal clusters

**Figure 4:**
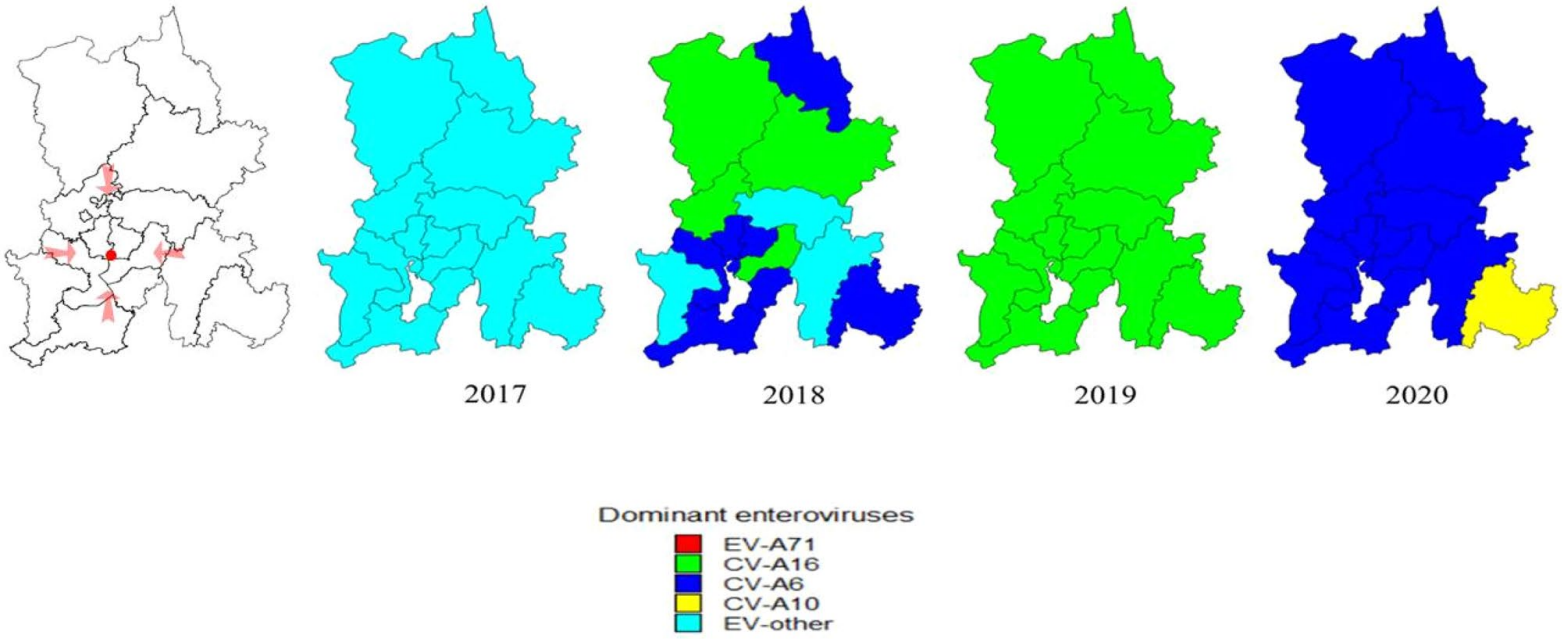
The spatial distribution map of other enteroviruses as dominant enteroviruses of districts and counties in Kunming, 2017–2020. The red dot shows the location of Kunming Children's Hospital, which is located at the junction of the main urban area. In 2017, the areas with a high proportion of other enteroviruses as dominant enteroviruses were Shilin (100%), Yiliang (94.7%), Anning (89.3%), Chenggong (85.1%) and Luquan (79.0%). In 2018, CV-A16 was mainly prevalent in Luquan (43.5%), Xundian (39.3%), Fumin (37.6%) and Guandu (33.2%); CV-A6 was mainly prevalent in Shilin (60.0%), Dongchuan (49.5%), Jinning (45.8%), Chenggong (37.8%) and Wuhua (36.5%). In 2019, the areas with a high proportion of CV-A16 as dominant enteroviruses were Jinning (59.9%), Yiliang (55.8%), Chenggong (49.5%), Panlong (48.4%) and Anning (48.2%). In 2020, CV-A6 was mainly prevalent in Luquan (77.4%), Dongchuan (75.7%), Anning (74.5%), Fumin (74.1%) and Xundian (73.4%); CV-A10 was mainly prevalent in Shilin (50.0%), Songming (19.1%) and Yiliang (17.1%). This figure was drawn by MapInfo 10.0 spatial distribution software [url = http://www.i-mapinfo.com] MapInfo[/url].

During 2017–2020, the periodic peaks of HFMD showed spatial–temporal clusters, and different pathogens showed different spatial–temporal distributions (Fig. 4). In 2017, the areas with a high incidence of other enteroviruses were Shilin (100%), Yiliang (94.7%), Anning (89.3%), Chenggong (85.1%) and Luquan (79.0%). In 2018, the areas with a high incidence of CV-A16 were Luquan (43.5%), Xundian (39.3%), Fumin (37.6%) and Guandu (33.2%); the areas with a high incidence of CV-A6 were Shilin (60.0%), Dongchuan (49.5%), Jinning (45.8%), Chenggong (37.8%) and Wuhua (36.5%). In 2019, the areas with a high incidence of CV-A16 were Jinning (59.9%), Yiliang (55.8%), Chenggong (49.5%), Panlong (48.4%) and Anning (48.2%). In 2020, the areas with a high incidence of CV-A6 were Luquan (77.4%), Dongchuan (75.7%), Anning (74.5%), Fumin (74.1%) and Xundian (73.4%); the areas with a high incidence of CV-A10 were Shilin (50.0%), Songming (19.1%) and Yiliang (17.1%).

## Discussion

According to World Health Organization (WHO) reports, China is one of the countries with a high incidence of HFMD^15^. Since the 2008 outbreak, HFMD has been listed as a type C statutory notifiable infectious disease^16^ that need constant surveillance. In the eastern coastal cities of China, previous studies have been carried out and summarized some regional epidemiological characteristics of HFMD, including the prevalence among children under five years of age^17^, a higher incidence among local males, one peak in a year^18^, and EV-A71 as the most common causative pathogen^1,7,19^.

In recent years, research on HFMD has gradually been conducted in central and western cities in China, such as Sichuan and Xinjiang, and obvious regional epidemiological characteristics have been found, such as two peak epidemic seasons in a year^1^, and EV-A71 and CV-A16 as the most common etiological agents in local areas^7,21^. Kunming, as an inland city in Southwest China, has also continuously surveilled HFMD for over a decade. A few reports showed the research results of the local incidence of HFMD before 2017, and found that children aged 1–3 who lived in different areas were at the highest risk, the ratio of males to females was 1.5:1, a major peak occurred during April to June followed by an autumn peak during October to November, and EV-A71, CV-A16, and other enteroviruses were the most common causative pathogens^22^. These results reflected some regional epidemiological and etiological characteristics of HFMD in Kunming before the EV-A71 vaccination campaign, and played a certain role in prevention and control.

Since 2016, the inactivated monovalent EV-A71 vaccine has been administered nationwide in China, which substantially reduces EV-A71-associated HFMD by over 90%^23^. However, due to the lack of cross-protection from the vaccine and the high rate of gene mutations and recombinations in other serotypes^24^, the prevalence of HFMD remains high; for example, CV-A16 replaced EV-A71 in Guangzhou, Shenzhen and Xinjiang, and CV-A6 prevailed as the dominant serotype in Xiangyang and Sichuan^4,14^. From 2017 to 2020, approximately 600,000 doses of inactivated monovalent EV-A71 vaccines were administered by the local CDC in Kunming, and the incidence of EV-A71-associated HFMD decreased dramatically, from 10.4% in 2017 to only 1.3% in 2019. In 2017, other enteroviruses replaced EV-A71 as the predominant serotypes and accounted for 76.2%. However, CV-A6 and CV-A16 showed an upward trend from 2017 to 2019, with sizable outbreaks in 2018 (33.3%) and 2019 (47.2%), respectively. Meanwhile, the peak time was longer than that before vaccination with the EV-A71 vaccine; the major peak extended for two months from April to August, and the minor peak extended for four months from September to the following February (Fig. 2). This phenomenon is mainly due to the EV-A71 vaccine imparting selective pressure for these other serotypes to emerge at high proportions^25^ and the different enterovirus genotypes with dissimilar activity and transmission characteristics under the influences of complex social and climatic factors^7,26^, which can cause differing scales and peak patterns^27^.

In addition, after the COVID-19 outbreak, we found that the incidence of CV-A6 and CV-A10 increased substantially, and CV-A6 replaced CV-A16 as the predominant serotype and accounted for 70.8%. From the end of 2019, the minor peak of CV-A6 disappeared and plateaued until the following February. In 2020, CV-A6 only presented a single peak that continued from July to the following February. We speculate that the main reasons are as follows. First, EVA71, CV-A16, CV-A6 and other enteroviruses circulate in 2–3- or 3–4- year cycles. The year 2020 may have been the end of the epidemic cycle of CV-A16, whereas the epidemic cycle of CV-A6 was beginning in Kunming. Second, effective personal hygiene measures during the epidemic period, such as reducing physical contact, wearing masks and washing hands, effectively reduced the transmission routes of HFMD. Third, public places were closed and children with HFMD were completely isolated at home. Fourth, the surveillance of population flow has obviously affected the epidemic and outbreak cycle of different pathogens.

To further understand the evolutionary dynamics of HFMD enteroviruses and enhance vaccination strategies in Kunming, we presented a the spatial and temporal description of their epidemiological and etiological characteristics. Spatial–temporal scanning includes geographic information, corrects nonuniform population density in different places, makes up for the deficiencies in the simple epidemiological morbidity comparisons, and objectively and comprehensively evaluates abnormal increases and the incidence aggregation area in both time and space dimensions^28^.

From 2017 to 2020, by using spatial–temporal scanning, we analyzed the number of cases of HFMD mainly concentrated in Guandu, Xishan, Wuhua, Panlong and Chenggong, and the high-incidence HFMD areas were Guandu, Chenggong and Xishan. Both the number and incidence of HFMD cases showed outbreaks in 2018 and 2019 and continued to increase in Chenggong. Spatial aggregation means that the risk of HFMD is substantially higher in some areas than in others, probably because of greater residential and population density, which can lead to clustered infections. In addition, it is interesting to note that these areas are mainly concentrated near the Panlong River and Dianchi Lake, which indicates that the prevalence of HFMD may be associated with humidity, pollution or other factors related to the lake. Moreover, different pathogens have different spatial and temporal distributions, and which also occur for the same pathogen. In 2017, other enteroviruses were the predominant serotypes, and the areas with high proportions of enteroviruses were Shilin, Yiliang and Anning. In 2018, CV-A16 was mainly prevalent in Luquan, Xundian and Fumin; CV-A6 was mainly prevalent in Shilin, Dongchuan and Jinning. In 2019, CV-A16 was the predominant serotype, and the areas with high proportions were Jinning, Yiliang and Chenggong. In 2020, CV-A6 was the predominant serotype, and the areas with high proportions were Luquan, Dongchuan and Anning; CV-A10 was mainly prevalent in Shilin, Songming and Yiliang. Although the annual total incidence of HFMD is directly related to population density, the annual incidence of different enterovirus serotypes differs in different regions, which pose a great challenges to the epidemic prevention of HFMD. It was reported that epidemics of EV-A71 or CV-A16 circulate in a cyclical pattern every 2–3 years^29^. Throughout Kunming after EV-A71 vaccination, we noticed that other enteroviruses have decreased substantially since 2018 but have remained stable at 15–20% since 2019. CV-A16 gradually increased from 2018 and led to an outbreak in 2019. CV-A6 had a regional outbreak in 2018 and gradually increased in 2019. CV-A10 cases have increased since 2018. After the COVID-19 pandemic, cases of CV-A6 broke out throughout Kunming, cases of CV-A10 broke out regionally, and other enteroviruses remained stable at 20% and surpassed CV-A6. We found that the epidemics and outbreaks of CV-A6- and CV-A16- associated HFMD appeared alternately, and the cycle was only 1 year; CV-A10 and other enteroviruses were potentially associated risk pathogens for outbreaks of HFMD in the next cycle period, although their incidence was not high.

This study has several limitations that should be acknowledged. First, HFMD is asymptomatic and self-limiting; thus, some cases of HFMD may be ignored through the passive surveillance system, and the actual number of cases may have been underestimated. Second, we did not test for enterovirus serotypes beyond EV-A71, CV-A16, CV-A6, CV-A10 and other serotypes because they had not been dominant in the past based on other studies. Third, we analyzed the association between the spatial and temporal distribution of infectious diseases, but the spread of disease is influenced by a variety of factors, such as meteorological, socioeconomic, health resource and traffic variables. However, due to limited data access, these factors were not considered in this study.

## Conclusions

This study confirms that the spatial and temporal distribution of HFMD varies at different scales, and the incidence of different pathogens associated with HFMD has obvious regional differences and seasonal trends. Therefore, the research and development of multivalent combined vaccines, including those for EV-A71, CV-A16, CV-A6 and CV-A10, are urgently needed, which could cover over 90% of pathogens associated with HFMD in the southwestern region of China. In addition, due to the COVID-19 pandemic, the number of infected patients was considerably low for 2020. Therefore, taking proper preventive and protective measures could effectively control the incidence of HFMD-like diseases in Kunming and even nationwide (Supplementary Information).

## Supplementary Information

Supplementary Information.

## Data Availability

Some or all data, models, or code generated or used during the study are available from the corresponding author by request.

## References

[1] Puenpa J (2013). Hand, foot, and mouth disease caused by coxsackievirus A6, Thailand, 2012. Emerg. Infect. Dis..

[2] Saki T, Liao Q, Van B (2016). Hand, Foot, and mouth disease in China: Modeling epidemic dynamics of enterovirus serotypes and implications for vaccination. PLoS Med..

[3] Sumi A, Toyoda S, Kanou K (2017). Association between meteorological factors and reported cases of hand, foot, and mouth disease from 2000 to 2015 in Japan. Epidemiol. Infect..

[4] Yang B, Liu F, Liao Q (2017). Epidemiology of hand, foot and mouth disease in China, 2008 to 2015 prior to the introduction of EV-A71 vaccine. Euro Surveill..

[5] Qi H, Li Y, Zhang J (2020). Quantifying the risk of hand, foot, and mouth disease (HFMD) attributable to meteorological factors in East China: A time series modelling study. Sci. Total Environ..

[6] Liang YK, Li N, Yang JZ, Deng B, Xie RH, Shu S (2012). Epidemiologic characteristics of hand-foot-mouth disease in Guiyang between 2008 and 2010. Zhongguo Dang Dai Er Ke Za Zhi.

[7] Xing W, Liao Q, Viboud C, Zhang J, Sun J, Wu JT (2014). Hand, foot, and mouth disease in China, 2008–12: an epidemiological study. Lancet. Infect. Dis.

[8] Sun LM, Wu SL, Tan XH, Li H, Yang F, Zeng HR (2018). Epidemiological characteristics of Coxsackie virus A16 caused hand foot and mouth disease cases in Guangdong province, 2012–2016. Zhonghua Liu Xing Bing Xue Za Zhi.

[9] Li Y, Bao H, Zhang X, Zhai M, Bao X, Wang D (2017). Epidemiological and genetic analysis concerning the non-enterovirus 71 and non-coxsackievirus A16 causative agents related to hand, foot and mouth disease in Anyang city, Henan Province, China, from 2011 to 2015. J. Med. Virol..

[10] Chen Q, Xing XS, Wu Y, Liao QH, Liu GP, Jiang XQ (2017). Hand, foot and mouth disease in Hubei province, 2009–2015: an epidemiological and etiological study. Zhonghua Liu Xing Bing Xue Za Zhi.

[11] Li R, Liu L, Mo Z (2014). An inactivated enterovirus 71 vaccine in healthy children. N. Engl. J. Med..

[12] Zhu F, Xu W, Xia J (2014). Efficacy, safety, and immunogenicity of an enterovirus 71 vaccine in China. N. Engl. J. Med..

[13] Zhou Y, Li JX, Jin PF (2016). Enterovirus 71: a whole virion inactivated enterovirus 71 vaccine. Expert Rev. Vac..

[14] Zhang J (2019). Trend of epidemics and variation of pathogens of hand, foot and mouth disease in China: a dynamic series analysis, 2008–2017. Chin. J. Endemiol..

[15] Owino CO, Chu J (2019). Recent advances on the role of host factors during non-poliovirus enteroviral infections. J. Biomed. Sci..

[16] Jian C, Wu J, Xu Z (2014). Associations between extreme precipitation and childhood hand, foot and mouth disease in urban and rural areas in Hefei China. Sci. Total Environ..

[17] Yang S, Wu J, Ding C (2017). Epidemiological features of and changes in incidence of infectious diseases in China in the first decade after the SARS outbreak: an observational trend study. Lancet Infect. Dis..

[18] Wang J, Teng Z, Cui X (2018). Epidemiological and serological surveillance of hand-foot-and-mouth disease in Shanghai, China, 2012–2016. Emerg. Microbes Infect.

[19] Ooi MH (2010). Clinical features, diagnosis, and management of enterovirus 71. Lancet Neurol..

[20] Gopalkrishna V, Patil PR, Patil GP (2012). Circulation of multiple enterovirus serotypes causing hand, foot and mouth disease in India. J. Med. Microbiol..

[21] Yuwei W, Wei C, Meng H (2017). Epidemiology and etiology of hand, foot, and mouth disease in Fujian province, 2008–2014. Arch. Virol..

[22] Hongchao J, Zhen Z, Qing R (2021). The epidemiological characteristics of enterovirus infection before and after the use of enterovirus 71 inactivated vaccine in Kunming China. Emerg. Microbes Infect..

[23] Lin JY, Kung YA, Shih SR (2019). Antivirals and vaccines for enterovirus A71. J Biomed. Sci..

[24] Nicholson E, Piedra PA (2017). Local versus global enterovirus (EV) surveillance: A discussion for the need for active surveillance to guide EV-A71 vaccines. J. Infect. Dis..

[25] Meng XD, Tong Y, Wei ZN (2020). Epidemical and etiological study on hand, foot and mouth disease following EV-A71 vaccination in Xiangyang, China. Sci. Rep..

[26] Chen M, He S, Yan Q (2017). Severe hand, foot and mouth disease associated with Coxsackievirus A10 infections in Xiamen, China in 2015. Clin. Virol..

[27] Di P, Yue Ma, Yaqiong L (2020). Epidemiological and aetiological characteristics of hand, foot, and mouth disease in Sichuan Province, China, 2011–2017. Sci. Rep..

[28] Wang Y, Zhao H, Ou R (2020). Epidemiological and clinical characteristics of severe hand-foot-and-mouth disease (HFMD) among children: A 6-year population-based study. BMC Public Health.

[29] Mao Q, Wang Y, Shao J (2015). The compatibility of inactivated-enterovirus 71 vaccination with coxsackievirus A16 and poliovirus immunizations in humans and animals. Hum. Vaccin. Immunother..

